# Association between Catechol-O-Methyltrasferase Val108/158Met Genotype and Prefrontal Hemodynamic Response in Schizophrenia

**DOI:** 10.1371/journal.pone.0005495

**Published:** 2009-05-08

**Authors:** Ryu Takizawa, Mamoru Tochigi, Yuki Kawakubo, Kohei Marumo, Tsukasa Sasaki, Masato Fukuda, Kiyoto Kasai

**Affiliations:** 1 Department of Neuropsychiatry, Graduate School of Medicine, The University of Tokyo, Tokyo, Japan; 2 Health Service Center, The University of Tokyo, Tokyo, Japan; 3 Department of Psychiatry and Human Behavior, Graduate School of Medicine, Gunma University, Gunma, Japan; Chiba University Center for Forensic Mental Health, Japan

## Abstract

**Background:**

“Imaging genetics” studies have shown that brain function by neuroimaging is a sensitive intermediate phenotype that bridges the gap between genes and psychiatric conditions. Although the evidence of association between functional val108/158met polymorphism of the catechol-O-methyltransferase gene (COMT) and increasing risk for developing schizophrenia from genetic association studies remains to be elucidated, one of the most topical findings from imaging genetics studies is the association between COMT genotype and prefrontal function in schizophrenia. The next important step in the translational approach is to establish a useful neuroimaging tool in clinical settings that is sensitive to COMT variation, so that the clinician could use the index to predict clinical response such as improvement in cognitive dysfunction by medication. Here, we investigated spatiotemporal characteristics of the association between prefrontal hemodynamic activation and the COMT genotype using a noninvasive neuroimaging technique, near-infrared spectroscopy (NIRS).

**Methodology/Principal Findings:**

Study participants included 45 patients with schizophrenia and 60 healthy controls matched for age and gender. Signals that are assumed to reflect regional cerebral blood volume were monitored over prefrontal regions from 52-channel NIRS and compared between two COMT genotype subgroups (Met carriers and Val/Val individuals) matched for age, gender, premorbid IQ, and task performance. The [oxy-Hb] increase in the Met carriers during the verbal fluency task was significantly greater than that in the Val/Val individuals in the frontopolar prefrontal cortex of patients with schizophrenia, although neither medication nor clinical symptoms differed significantly between the two subgroups. These differences were not found to be significant in healthy controls.

**Conclusions/Significance:**

These data suggest that the prefrontal NIRS signals can noninvasively detect the impact of COMT variation in patients with schizophrenia. NIRS may be a promising candidate translational approach in psychiatric neuroimaging.

## Introduction

Schizophrenia is a complex psychiatric disease that is speculated to result from multiple genetic and environmental factors. Due to familial aggregation and high heritability, the involvement of genetic factors in the onset of schizophrenia is considered to be relatively large [1].

The gene for catechol-O-methyltransferase (COMT) that metabolizes released dopamine is one of the susceptibility genes located in chromosome 22q11.2 and has been repeatedly reported to be involved in schizophrenia [2]–[5]. Many association studies have indicated the involvement of a functional polymorphism (Met108/158Val) (*rs4680*) with the slight increase in risk for schizophrenia [6], [7]. However, other studies have reported no association [8], [9] and there are several other putatively functional single nucleotide polymorphisms (SNPs) that have been implicated [10], [11]. Thus, the association studies are yet to converge on a consistent finding.

Meanwhile, in humans, there is a COMT genotype (Met108/158Val) where a fourfold reduction of COMT enzyme activity is derived from the substitution of a valine (Val) by a methionine (Met) [12]. Moreover, the activity of the COMT enzyme is assumed to modulate dopamine levels in the prefrontal cortex (PFC) [13], [14]. A few reports suggested the inverted-U-shape phenomenon in the relationship between dopamine level and brain function. The phenomenon is based on the hypothesis that there would be an optimal prefrontal level of dopamine for efficient physiological signaling and optimal performance, and that PFC function was impaired when dopaminergic nervous transmission was either too far above or below normal levels [15]–[17]. Some studies indicated that the COMT Val/Met genotype has an effect on the response to antipsychotic drugs, because dopamine release in the PFC is enhanced by antipsychotic medication [14], [18]. Other studies suggested that a subpopulation of patients with the COMT Met genotype might respond more favorably to antipsychotic treatment [19], [20]. Also, these studies showed that functional MRI changes associated with good cognitive response to antipsychotic drugs were predicted on the basis of the COMT genotype. It would be ideal for clinical application and translational approach if neuroimaging techniques could be used to determine the intermediate phenotype to help predict such effects before starting the treatment.

From the perspective of clinical application and translational approach, the use of a less psychologically and physically demanding neuroimaging method is necessary. Conducting neuroimaging that requires less restraint on movement and less time is better for patients, particularly psychiatric patients. Multichannel near-infrared spectroscopy (NIRS), a relatively new functional neuroimaging technology, is one of the potent candidates that is restraint-free, easy-to-use, portable and noninvasive. Furthermore, the NIRS equipment is relatively inexpensive and with almost no running cost except for the electricity charges during use. Some neuroimaging techniques have limitations in that the measurement makes the subjects uncomfortable in an unnatural setting, e.g., lying in a supine position in a narrow gantry and fixing the head during the entire examination. In contrast, NIRS measurement under natural conditions, in a sitting position in a well-lit room, may overcome these limitations. We have sought to establish one of the best cognitive activation tasks for NIRS, and previously found that verbal fluency is the most reliable task showing prominent and wide-spread frontotemporal activation in normal subjects that can be easily differentiated from that in patients with schizophrenia [21], [22]. The time needed to set up the apparatus is usually less than 5 minutes including the audiovisual on-screen instructions, and our version of the verbal fluency task takes less than 3 minutes, which is less demanding for the participants. Furthermore, if imaging genetics research demands a larger and wider variety of samples in the near future, NIRS has great potential as a neuroimaging modality to detect cortical function with ease and speed.

Multichannel NIRS enables the noninvasive and bedside measurement of the spatiotemporal characteristics of brain function, which are assumed to reflect regional cerebral blood volume. Using NIRS, typical cortical activations show not only a deoxy-hemoglobin concentration ([deoxy-Hb]) decrease that is the putative main source of blood-oxygenation-level-dependent (BOLD)-contrast increase as measured by fMRI, but also a relatively larger oxy-hemoglobin concentration ([oxy-Hb]) increase. The usefulness and limitations of NIRS have been discussed widely in previous literatures [23]–[26]. NIRS has been used to assess brain function in many psychiatric disorders, including schizophrenia, bipolar disorder, depression, dementia, posttraumatic stress disorder, and pervasive developmental disorders[21], [22], [27]–[30]. Considering the effect of the COMT genotype on the response to antipsychotic drugs, if an association is found between NIRS signals and functional polymorphisms in the COMT gene, NIRS signals could be potentially used as a biological marker to aid in the evaluation and prediction of response to medication.

The purpose of this study was to use a noninvasive and less-demanding NIRS instrument with a wide coverage of the prefrontal cortex to investigate the association between COMT genotype and prefrontal hemodynamic response in healthy controls and patients with schizophrenia, and to test whether NIRS could be a promising candidate for future translational approach in psychiatry neuroimaging.

## Methods

### Participants

Participants were 45 stably maintained outpatients with chronic schizophrenia and 60 healthy controls matched for age and gender (Table 1). The demographic results between healthy controls and patients with schizophrenia showed the significant difference in education, socioeconomic status, estimated premorbid IQ, and task performance. Because matching for all the factors lead to the drastic reduction in number of the subjects in each genotype we did not match for these factors. Instead, we gave priority to match these factors for the two genotypes in each diagnostic group. All the participants were native Japanese speakers and right-handed (>70) according to Oldfield's Edinburgh Inventory [31].

**Table 1:** Clinical characteristics of study groups.

	Patients with Schizophrenia (N = 45)					Healthy controls (N = 60)					Group difference
	Met carriers (N = 25)		Val/Val (N = 20)		*t*-test	Met carriers (N = 30)		Val/Val (N = 30)		*t*-test	t-test
	Mean	SD	Mean	SD	p-value	Mean	SD	Mean	SD	p-value	p-value
Age, years	41.0	9.5	41.5	11.9	.89	37.2	12.6	37.7	13.6	.86	.12
Gender, women/men	13/12		12/8		.59*	14/16		12/18		.60*	.22*
Education, years	14.0	2.6	15.0	2.7	.26	16.2	3.0	16.7	2.9	.49	<.00
Socioeconomic status (SES)	3.5	1.2	3.6	1.1	.72	1.8	0.7	1.6	0.6	.30	<.00
Estimated premorbid IQ	99.4	12.4	101.7	13.3	.54	105.7	12.4	109.0	7.2	.22	.00
Task performance	13.7	4.3	14.8	5.9	.48	16.6	4.4	17.2	4.6	.63	.01
Age at onset, years	28.2	9.2	22.1	5.3	.09	NA		NA			
Duration of illness, years	12.8	7.7	17.3	12.4	.16	NA		NA			
PANSS											
positive	15.9	5.5	15.8	4.7	.93	NA		NA			
negative	20.8	6.9	21.7	6.1	.40	NA		NA			
general psychopathology	36.5	8.6	38.2	6.4	.47	NA		NA			
Medication											
Chlorpromazine equivalent dose, mg/day	582.5	483.5	901.4	693.5	.09	NA		NA			
Diazepam equivalent dose, mg/day	10.7	10.9	6.9	10.2	.24	NA		NA			
Biperiden equivalent dose, mg/day	3.1	2.3	4.0	2.2	.25	NA		NA			

Abbreviations: IQ, Intelligence Quotient; PANSS, Positive and Negative Symptom Scale; NA, not applicable.

* Chi-square test was used for testing group difference in gender distribution. Otherwise, *t*-test was used.

The patients were recruited from among outpatients of the University of Tokyo Hospital. The diagnosis of schizophrenia was made through the Structured Clinical Interview for DSM-IV Axis I Disorders (SCID) [32] by an experienced psychiatrist (K.K.). For screening healthy controls, SCID non-patient edition (SCID-NP) was used. On the same day as the NIRS experiment, psychiatric symptoms were evaluated by one psychiatrist (K.K.) using the Positive and Negative Syndrome Scale (PANSS) [33], without knowledge of the NIRS data. At the time of the study, the patients with schizophrenia were on medication with antipsychotics and/or anxiolytics and/or antiparkinsonian agents. Socioeconomic status (SES) was assessed using the Hollingshead Index of social position [34]. Premorbid IQs were estimated using the Japanese version of National Adult Reading Test [35] (Table 1).

The exclusion criteria for both groups were neurological illness, traumatic brain injury with any known cognitive consequences or loss of consciousness for more than 5 minutes, a history of electroconvulsive therapy, and alcohol/substance abuse or addiction. An additional exclusion criterion for the healthy control group was a history of psychiatric disease in themselves or a family history of axis I disorder in their first-degree relatives.

### Ethics

The ethics committee of the University of Tokyo Hospital approved this study (receipt number: 630). All the subjects gave written informed consent in accordance with the Declaration of Helsinki after a complete explanation of the study.

### Cognitive Activation task

The cognitive activation task, which was described previously [21], [22], [28], included a 30-sec pretask baseline, a 60-sec verbal fluency task (letter version), and a 70-sec posttask baseline while sitting on a chair. For the pre- and posttask baseline periods, the subjects were instructed to repeat Japanese vowels (/a/, /i/, /u/, /e/ and /o/) out loud throughout the period. This was intended to correct the fluency task data for activation due to vocalization.

During the verbal fluency task period, they were instructed to generate as many Japanese words beginning with a designated syllable as possible. We modified the commonly used 60-sec verbal fluency task in which one initial syllable is presented. In this study, three initial syllables (first, /to/, /a/, or /na/; second, /i/, /ki/, or /se/; third, /ta/, /o/, or /ha/) were presented in order, which was counterbalanced among the subjects and changed every 20 sec during the 60-sec task to reduce the time during which the subjects remained silent or gave up the task. An auditory cue alerted the subjects to the start and end of each task and when the syllable was changed. The total number of correct words generated during the 60-sec activation period was defined as a measure of task performance (Table 1).

### NIRS measurement

The 52-channel NIRS machine (ETG-4000, Hitachi Medical Co., JAPAN) measures relative changes in [oxy-Hb] and [deoxy-Hb] using two wavelengths (695 and 830 nm) of infrared light based on the modified Beer-Lambert law [36]. In this continuous-wave NIRS system, these [Hb] values include a differential pathlength factor (DPF); therefore, the unit of the NIRS measurement is mM?mm. The distance between pairs of source-detector probes was set at 3.0 cm and we defined each measuring area between pairs of source-detector probes as one ‘channel’. It is assumed that a machine, in which the source-detector spacing is 3.0 cm, measures points at 2–3 cm depth from the scalp, that is, the surface of the cerebral cortex [37]. The probes of the NIRS machine were fixed with thermoplastic 3×11 shells, with the lowest probes positioned along the T4-Fpz-T3 line according to the international 10–20 system used in electroencephalography. The 52 measurement areas are labeled ch1–ch52 from the right-posterior to the left-anterior. This arrangement of the probes can measure [Hb] from the bilateral prefrontal (approximately dorsolateral [Brodmann's area (BA) 9, 46], ventrolateral [BA 44, 45, 47], and frontopolar [BA 10]) and superior temporal cortical surface regions. The correspondence of the probe positions and the measurement areas on the cerebral cortex was confirmed by superimposing the measurement positions on a magnetic resonance image of a three-dimensionally reconstructed cerebral cortex of a representative subject among the healthy controls, and the correspondence was also supported by a multisubject study of anatomical cranio-cerebral correction via the international 10–20 system [38], [39].

The time resolution of NIRS was set at 0.1 sec. The NIRS signal changes were analyzed using the first-order correction to exclude task-unrelated changes during the verbal fluency task; Because the NIRS signal was sometimes unstable at the start of the pretask, the pretask baseline was determined as the mean across the last 10 sec of the pretask period, and the posttask baseline was determined as the mean across the last 5 sec of the posttask period, and a linear fitting was performed on the basis of data between the two baselines. The fluctuations of NIRS signals were known to be related to such physiological activities as the systemic arterial pulse oscillations (∼1 Hz) and respiration (0.2–0.3 Hz) [24]. Thus, moving average methods were applied to remove short-term motion artifacts and to correct such fluctuations in the analyzed data (moving average window: 5 sec). Grand mean waveforms averaged across subjects were created separately for each type of [Hb] and for each group. Because the moving average methods cannot be used to correct all the artifacts, according to the algorithm for quantitatively evaluating the artifacts (in supplementary material of Takizawa *et al.* [22]), we performed a fully automatic rejection of data with artifacts separately for each channel, i.e., the number of averaged subjects varied across channels (schizophrenia: N = 29–45 [mean, 39.5; SD, 3.4]; healthy subjects: N = 40–60 [mean, 52.0; SD, 5.5]; percentage: schizophrenia, 87.7%; healthy subjects, 86.7%, n.s.).

### Genotyping

Genomic DNA was extracted from leukocytes using a standard method. Genotyping of the COMT Val108/158Met polymorphism was performed using the ABI PRISM 7900HT Sequence Detection System (Applied Biosystems, California, USA).

As shown in the results section, the sample size of the Met/Met genotype was too small to provide sufficient statistical power to draw a conclusion. Thus, the three genotypes in the COMT gene were classified into two genotype subgroups according to the effect on the catalysis of catecholamines: the genotypes with the Met allele (we call ‘Met carriers’) versus the Val/Val genotype (we call ‘Val/Val individuals’). The Met allele genotype of the COMT gene is associated with reduced catabolism of dopamine. The two genotype subgroups were matched for age, gender, IQ, and task performance in each diagnostic group (Table 1). This study was performed in ethnically homogeneous samples (only of Japanese descent).

### Statistical methods

For data analysis using parametric statistical tests, obtained [Hb] data from each channel were averaged across the two time segments (pretask baseline and task period). We focused on [oxy-Hb] here, since [oxy-Hb] increase is assumed to more directly reflect cognitive activation than [deoxy-Hb] decrease as shown by a stronger correlation with the BOLD signal measured by fMRI [40] and by the results of animal studies [41], although the results of the analysis of [deoxy-Hb] were also presented.

First, in healthy controls, to confirm the increase associated with the verbal fluency task, the mean [Hb] for the pretask baseline period and that for the task period were compared at each channel using Student's paired *t*-test (two-tailed). False discovery rate (FDR) correction for multiple comparisons [52 channels] was applied. We set the value specifying the maximum FDR to 0.05, so that there are no more than 5% false positives on average [42].

Second, *t*-tests for differences in potential confounding factors, such as age, gender, education, premorbid IQ, task performance, clinical symptoms, and dose of medication were performed between the two COMT genotype subgroups in healthy controls and in patients with schizophrenia.

Third, we thought it ideal to analyze the data by repeated-measures ANOVA with 52 channels as a within-subjects factor, group (normal controls versus patients with schizophrenia) and genotype (Met carriers versus Val/Val individuals) as between-subjects factors. However, we excluded the data from the channels that show undesirable artifacts, instead of rejecting all data of the subject. If we used ANOVA, the data on a considerable number of subjects should be omitted. In fact, the ANOVA permitted including only 17 subjects (3 patients with schizophrenia and 14 healthy controls) out of original 105 (45 patients with schizophrenia and 60 healthy controls). Accordingly, the reduced statistical power did not yield significant genotype-by-group interaction in the ANOVA (F[1], [13] = 0.52, p = 0.48). Thus, we stratified the participants into normal controls and patients with schizophrenia, and then performed t-tests in each channel between the two genotypes for each group and conducted the correction for multiple comparisons using false discovery rate (FDR).

To analyze the pattern of signal changes along the time course with not only the amplitude but also the initial rise rate of hemodynamic response, the first 5-sec slope and mean [Hb] changes during the 60-sec task period between the two COMT genotype subgroups were compared in each diagnostic group. NIRS signal changes were compared between the two genotypes at each channel by two-tailed Student's *t*-test. P-values were adjusted by FDR correction for multiple comparisons [p<FDR 0.05; 52 channels]. Two-tailed analysis was used because task-load-dependent hypo- or hyperperfusions of the prefrontal cortex in patients with schizophrenia relative to healthy controls were reported previously [43], [44]. When channels were considered significant, we showed the effect size (cohen's d) and 95% confidence interval (CI). Statistical analysis was performed using SPSS 10.1.3J software (Tokyo, Japan).

## Results

### Genotype determination

The genotypic distributions of the three genotypes in the COMT genes are as follows: healthy controls, Met/Met 5 (8.3%), Val/Met 25 (41.7%), Val/Val 30 (50.0%); patients with schizophrenia, Met/Met 4 (8.9%), Val/Met 21(46.7%), Val/Val 20 (44.4%). The distributions of the three COMT genotypes were not significantly different from those expected according to the Hardy-Weinberg equilibrium in either diagnostic group.

### Test for significance of [Hb] change during activation period relative to baseline

In the healthy controls, a significant increase in [oxy-Hb] occurred during the task period relative to the pretask baseline at all the 52 channels (FDR corrected p; 0.0001–0.003) and a significant decrease in [deoxy-Hb] occurred during the task period relative to the pretask baseline at 39 channels (ch9, ch13–16, ch18–20, ch22–52; FDR-corrected p: 0.0001–0.038); thus, the prefrontal hemodynamic cortical activation due to the cognitive task was confirmed. In the present NIRS study, the verbal fluency task recruited widespread regions of the prefrontal cortical surface area and superior temporal regions, which is in accordance with previous studies using fMRI and PET [45], [46].

### Potential confounding factors

Potential confounding factors such as age, gender, education, premorbid IQ, task performance (total number of correct words generated), clinical symptoms, and dose of medication were not significantly different between the two COMT genotype subgroups in either diagnostic group (Table 1). The first 5-sec [oxy-Hb] slope and mean [oxy-Hb] change were not different between women and men for either diagnostic group (two-tailed *t*-test: p>FDR 0.05). In the healthy controls, the first 5-sec [oxy-Hb] slope and mean [oxy-Hb] change were not significantly correlated with any potential confounding factors (age, education, premorbid IQ, task performance) at any channel (Pearson's correlation coefficient: p>FDR 0.05). In the patients with schizophrenia, the 5-sec [oxy-Hb] slope and mean [oxy-Hb] change were also not significantly correlated with any potential confounding factors (age, education, premorbid IQ, task performance, PANSS scores, chlorpromazine equivalent dose, biperiden equivalent dose, and diazepam equivalent dose) at any channel (Pearson's correlation coefficient: p>FDR 0.05). The statistical conclusions were the same for [deoxy-Hb].

### Group comparisons in [oxy-Hb] response for significant genotype effects

First, we sought to confirm a significant difference in [oxy-Hb] response between the two diagnostic groups as shown in previous NIRS literature [21], [22]. As predicted, the schizophrenia patients showed a significantly lower [oxy-Hb] increase during the 60-sec task period than the healthy controls at 50 channels (all channels except for ch33, ch44; FDR-corrected p: 0.0001–0.048). We then investigated the differences between the two COMT genotype subgroups in each diagnostic group.

**Figure 1:**
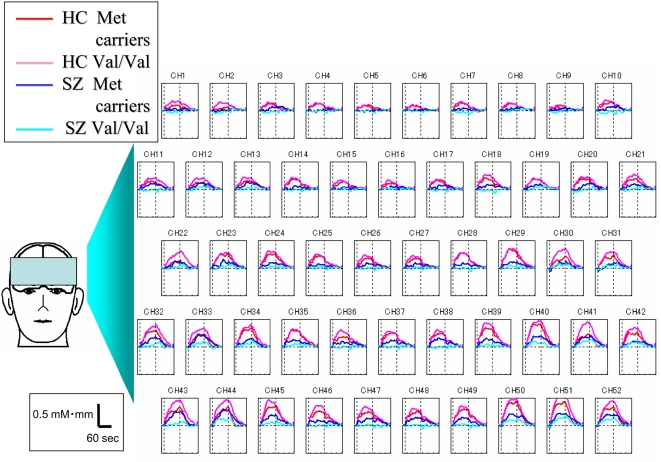
Relationship between COMT genotype and grand average waveforms in healthy controls (HC) and patients with schizophrenia (SZ). [oxy-Hb] changes during cognitive activation are presented as grand average waveforms in 52 channels in red (Met carriers among HC), pink (Val/Val individuals among HC), blue (Met carriers among SZ) and light-blue (Val/Val individuals among SZ) lines, respectively.

In the healthy controls, the first 5-sec [oxy-Hb] slope and mean [oxy-Hb] changes during the 60-sec task periods showed no significant difference at any channel between the Met carriers and the Val/Val individuals (p>FDR 0.05) (Figure 1).

**Figure 2:**
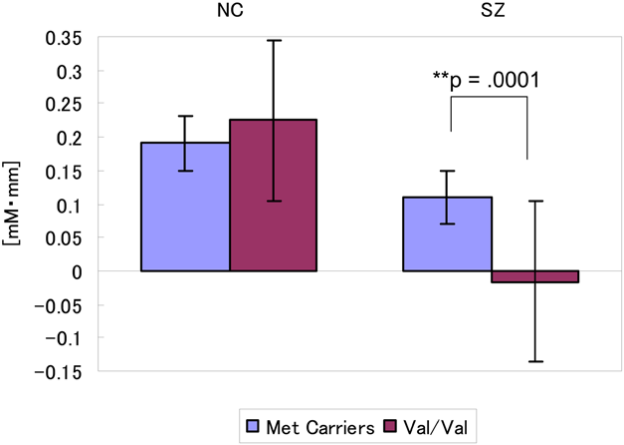
Relationship between COMT genotype and [oxy-Hb] change in healthy controls (HC) and patients with schizophrenia (SZ). The mean [oxy-Hb] change in the Met carriers and the Val/Val genotype subgroups among HC and SZ are indicated in a representative channel (Channel 38; left frontopolar PFC region). The bars show standard error of the mean.

**Figure 3:**
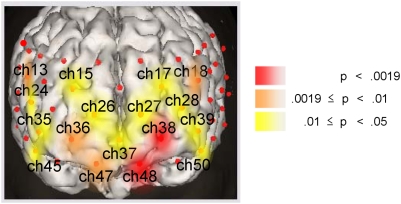
Prefrontal cortical distribution of significant effect of the COMT genotype on [oxy-Hb] changes in patients with schizophrenia. The channels with a significant genotype difference (P≤0.05) between the Met carriers and the Val/Val individuals among patients with schizophrenia are indicated by colored areas. To illustrate the gradation in P value over the prefrontal cortical surface area, channels with FDR-corrected P<0.0019 are colored in red, 0.0019≤P<0.01 in orange, and P≤0.05 in yellow. These areas approximately correspond to the bilateral frontopolar (BA 10), dorsolateral (BA 9 and 46), and ventrolateral (BA 44 and 45) prefrontal regions.

In the patients with schizophrenia, the [oxy-Hb] increases in the Met carriers were significantly greater than those in the Val/Val individuals at two channels that corresponded approximately to the frontopolar PFC regions (ch38 (p = 0.00114; d = 1.07; 95%CI 0.40, 1.70; Met carriers: N = 23, Val/Val individuals: N = 19) and ch48 (p = 0.00094; d = 1.08; 95%IC 0.39, 1.72; Met carriers: N = 22, Val/Val individuals: N = 18); FDR-corrected p: 0.0001–0.0019) (Figure 2). Additionally, a few more channels that showed a trend-level difference (uncorrected p<0.05 but not remaining significant after FDR correction) were located approximately in the bilateral frontopolar ([FP]), dorsolateral ([DL]) and ventrolateral ([VL]) regions (uncorrected p<0.01; [FP] ch 36 (d = 0.83; 95%IC 0.19, 1.43), ch47 (d = 0.93; 95%IC 0.26,1.56), [DL] ch13 (d = 1.08; 95%IC 0.29, 1.80), ch18 (d = 0.84; 95%IC 0.19,1.46): uncorrected p<0.05; [FP] ch26 (d = 0.63; 95% 0.01, 1.23), ch27 (d = 0.73; 95%CI 0.09, 1.34), ch37 (d = 0.70; 95%CI 0.04, 1.33), [DL] ch15 (d = 0.85; 95%CI −0.01, 1.65), ch17 (d = 1.38; 95%CI 0.20, 2.43), ch24 (d = 0.65; 95%CI 0.03, 1.25), ch28 (d = 0.69; 95%CI 0.04, 1.30), ch35 (d = 0.68, 95%CI 0.05, 1.29), ch39 (d = 0.66; 95%CI 0.04, 1.26), [VL] ch45 (d = 0.83; 95%CI 0.12, 1.50), ch50 (d = 0.66; 95%CI −0.01, 1.30)) over the prefrontal cortex (Figure 3). However, the first 5-sec [oxy-Hb] slopes were not significantly different between the two genotype subgroups (P>FDR .05).

We also performed additional confirmatory analyses in patients with schizophrenia, because the chlorpromazine equivalent dose (CPZ) was not well matched (Met carriers: mean 582.5, SD 483.5; Val/Val individuals: mean 901.4, SD 693.5; p = 0.09, d = −0.54, 95%IC −1.13, 0.06). First, the results of a one-way analysis of covariance (ANCOVA) with CPZ as a covariate in the two significant channels (ch38 and ch48) also showed a significant main effect of the COMT genotype (ch38: F = 9.49, df = 1, p = 0.004; ch48: F = 12.39, df = 1, p = 0.001). Second, using the median value of CPZ (639.8 mg/day), we stratified the Val/Val individuals among the patients with schizophrenia into those with below median value (N = 10; 363.6±162.2 mg/day) and those with above median value (N = 10; 1439.3±588.5 mg/day). The [oxy-Hb] changes between the two subgroups of Val/Val individuals were not different in any channel (p's>0.2). Furthermore, the [oxy-Hb] changes in the Val/Val individuals with below median value of CPZ were significantly lower than those in the Met carriers ([FP] ch27: p = 0.02297; ch36: p = 0.02364; ch38: p = 0.00473; ch47: p = 0.00151; ch48: p = 0.00318; [DL] ch13: p = 0.04059; ch35: p = 0.04444; [VL] ch45: p = 0.01473).

### Group comparisons in [deoxy-Hb] response for significant genotype effects

The schizophrenia patients were associated with a significantly smaller [deoxy-Hb] decrease during the 60-sec task periods than healthy controls at 18 channels (ch18–19, ch24, ch28–29, ch31, ch33–34, ch36–40, ch42, ch45, ch48–50; FDR-corrected p: 0.0001–0.017).

The first 5-sec slope and mean changes of [deoxy-Hb] during the 60-sec task periods showed no significant difference at any channel between Met carriers and Val/Val individuals in either diagnostic group (p>FDR 0.05).

### Confirmatory Analysis between Val/Met and Val/Val genotype subgroups

To clarify the impact of COMT genotype at great length, we also conducted the confirmatory analysis to drop the Met/Met subjects from the analyses (patients with schizophrenia: Val/Met: N = 21, Val/Val: N = 20; Healthy controls: Val/Met: N = 25, Val/Val: N = 30).

When we dropped the Met/Met subjects from the analyses, potential confounding factors such as age, gender, education, premorbid IQ, task performance (total number of correct words generated), clinical symptoms, and dose of medication were also not significantly different between the two COMT genotype subgroups (Val/Met vs. Val/Val) in either diagnostic group. In particular, the CPZ equivalent in the patients with schizophrenia was not significantly different between the Val/Met genotype subgroup (594.1±464.2 mg/day) and the Val/Val genotype subgroup (901.4±693.5 mg/day) (p = 0.11, n.s.).

In the healthy controls, the first 5-sec [oxy-Hb] slope and mean [oxy-Hb] changes during the 60-sec task periods showed no significant difference at any channel between the Val/Met and Val/Val individuals (p>FDR 0.05).

In the patients with schizophrenia, the first 5-sec [oxy-Hb] slope and mean [oxy-Hb] changes during the 60-sec task periods also showed no significant difference at any channel between the Val/Met and Val/Val individuals (p>FDR 0.05). However, in a similar manner with the difference of mean [oxy-Hb] change between the Met carriers and Val/Val genotype subgroups, a few channels that showed a trend-level difference (uncorrected p<0.05 but not remaining significant after FDR correction) were located approximately in the bilateral frontopolar ([FP]), dorsolateral ([DL]) and ventrolateral ([VL]) regions. (uncorrected p<0.01; [FP] ch27 (p = 0.0019), ch38 (p = 0.0020), ch48 (p = 0.0017), [DL]:ch11 (p = 0.0085); ch13 (p = 0.0081); ch18 (p = 0.0095): uncorrected p<0.05; [FP]: ch26 (p = 0.0457), ch36 (p = 0.0126), ch47 (p = 0.0103), [DL]: ch3 (p = 0.0138), ch8 (p = 0.0241), ch10 (0.0271), ch24 (p = 0.0184), ch28 (p = 0.0271), [VL]: ch 32 (p = 0.0315), ch35 (p = 0.0261), ch45 (p = 0.0260) over the PFC.

The first 5-sec slope and mean changes of [deoxy-Hb] during the 60-sec task periods showed no significant difference at any channel between Met carriers and Val/Val individuals in either diagnostic group (p>FDR 0.05).

## Discussion

Using a 52-channel NIRS with a wide coverage over the prefrontal cortical surface area, we showed that the prefrontal hemodynamic response during a verbal fluency task was significantly associated with COMT genotype in patients with schizophrenia, but not in healthy controls. The increase in [oxy-Hb] in the COMT Met carriers during the verbal fluency task was significantly greater than that of COMT Val/Val individuals in the PFC of patients with schizophrenia, but these differences were not found to be significant in healthy controls. In this COMT genotype comparison, potential confounding factors such as age, gender, education, premorbid IQ, task performance, clinical symptoms, and dose of medication were matched between the two genotype subgroups in both diagnostic groups, and therefore were not likely to account for the effect of the COMT genotype on the prefrontal hemodynamic response of patients with schizophrenia. Furthermore, the use of the 52-channel NIRS in this study revealed that the significant association between the COMT genotype and prefrontal hemodynamic activation in patients with schizophrenia was most profound in the frontopolar PFC (BA10; also known as anterior or rostral PFC) and had a gradation over the prefrontal cortical area tapering from the frontopolar PFC to the more lateral PFC (dorsolateral and ventrolateral PFC) (Figure 3).

To our knowledge, this is the first NIRS report that implicated the impact of COMT genotype on prefrontal cortical function in schizophrenia. These data, consistent with findings from previous fMRI, ERP (P50, P300), PET and neuropsychological studies [7], [47]–[53], suggest that the COMT Met allele may have a beneficial effect on prefrontal function in schizophrenia. These results lead to the suggestion that prefrontal activity assessed by NIRS may offer promise as a noninvasive clinical tool for evaluating the spatiotemporal characteristics of prefrontal function in schizophrenia.

### No effect of COMT genotype in healthy controls

**Figure 4:**
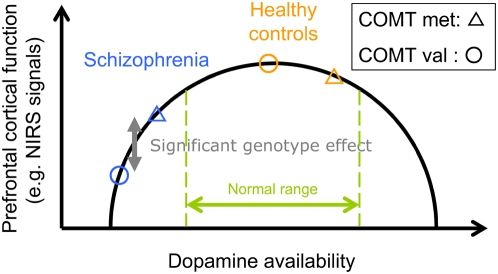
The hypothetical diagram of the inverted-U shaped relationship between dopamine availability and prefrontal cortical function.

Dopamine plays a critical role in modulating normal PFC function, and the modulation of dopamine levels via catabolism by COMT is considered to be important in the PFC, relative to the striatum, because it was reported that the expression of dopamine transporters in the PFC is less than that in the striatum [54]. As the Val allele facilitates dopamine metabolism, dopamine in the PFC of the individuals with the COMT Val genotype is less than that with the Met genotype, which leads to functional attenuation of the dopaminergic nervous system in individuals with the COMT Val genotype. Therefore, we need to discuss why we did not find a significant association between the COMT genotype and prefrontal function in healthy controls, which is similar to the results of a previous imaging genetics study on the COMT genotype in a Japanese population [55]. One possibility would be that healthy controls with the COMT Val/Val genotype might have other factors to compensate for the attenuation of the dopamine level via catabolism by COMT in the PFC, because the dopaminergic nervous system and PFC function might be modulated by not only COMT SNP variation. Considering the hypothesis on the inverted-U-shaped relationship between dopamine levels and PFC function [16], [56], it might be that healthy controls regardless of Met/Val polymorphism in the COMT genotype are almost all situated in the vicinity of the optimum level on the inverted-U curve of the prefrontal cortical function (Figure 4). Furthermore, three channels that showed a trend-level difference (uncorrected p<0.05 but not remaining significant after FDR correction) were located approximately in the left dorsolateral ([DL]) and bilateral ventrolateral ([VL]) regions ([DL]: ch 30 (d = −0.55; 95%IC −1.17, 0.10) [VL]: ch22 (d = −0.52: 95%IC −1.08, 0.06), ch 41 (d = −0.91; 95%IC −1.48, −0.30)), which showed that the prefrontal hemodynamic response of the Val/Val individuals was greater than that of the Met carriers among the healthy controls. These inverse slight COMT genotype effects (with nonsignificance but more than medium effect size) in normal controls suggested the possibility that individuals with the Met allele might have dopamine levels that are not necessarily more functionally optimal than those of the Val/Val individuals under normal conditions. In contrast, the inverted-U curve may be shifted in schizophrenia, possibly resulting in a larger effect of the COMT genotype on the prefrontal cortical function as measured by NIRS (Figure 4).

### Effect of COMT genotype on prefrontal subregions

In previous fMRI and PET studies that employed the n-back task [7], [49], [53], a significant effect of the COMT genotype in patients with schizophrenia was reported in the dorsolateral and ventrolateral PFC. In this study, the prominent impact of the COMT in patients with schizophrenia was found in the frontopolar PFC, whereas there was also a significant trend (uncorrected p<0.01) in the dorsolateral and ventrolateral PFC. Possible explanations for this discrepancy may be as follows. First, there might be a task-related difference in prefrontal cortical activation between verbal fluency and n-back. The verbal fluency task not only concurrently requires working memory capacity to hold the already-generated words but also requires retrieval of items from long-term memory storage and inhibition of inappropriate response [57]. Thus, the verbal fluency task might recruit broader regions including the frontopolar prefrontal cortical area than n-back, which requires a more simple working memory capacity [45].

Second, consistent with previous neuroimaging studies, larger [oxy-Hb] increase and concurrent large [deoxy-Hb] decrease during the verbal fluency task were detected in the dorsolateral and ventrolateral PFC using NIRS [21], [22], [28]. In contrast, however, the frontopolar hemodynamic response showing large [oxy-Hb] increase but slight [deoxy-Hb] decrease may be an activation pattern that is relatively difficult to detect using BOLD-contrast fMRI, because the decrease in paramagnetic [deoxy-Hb] is the primary source of BOLD-contrast increases (Table 2). Furthermore, because the frontopolar cortex is located in the vicinity of air-filled spaces of the nasal cavity, the corresponding magnetic susceptibility differences at the air–tissue or bone–tissue interface result in severe distortions and regional signal losses in long-TE gradient-echo images, particularly for ultrafast imaging techniques such as echo-planar imaging at a high magnetic field. Also, several studies of NIRS and fMRI measurements with the same individuals undergoing the same task suggested that the two signals were in good agreement [58]. Thus, the ability to observe both [oxy-Hb] and [deoxy-Hb] change in the frontopolar region may be an advantageous feature of NIRS.

**Table 2:** Pattern diagram of NIRS signal activations by PFC regions.

	Dorsolateral and Ventrolateral PFC	Frontopolar PFC
[oxy-Hb]	↑↑↑	↑↑
[deoxy-Hb]	↓↓	↓∼→

Third, the frontopolar PFC (BA10) has evolved to become large and specialized to play a role in high-order integrative prefrontal function, which integrates the outcomes of two or more separate cognitive operations involving dorsolateral and ventrolateral PFC functions in the pursuit of a higher behavioral goal [59], [60]. Interestingly, the Met allele of the COMT genotype appears to be unique to humans, because it has not been found in nonhuman primates [61]. The evolutionary implications in association with high-order cognitive function in human hierarchical PFC might explain the prominent genotype effect found in the frontopolar PFC (BA10) [60]. In this study, the reduced NIRS signal changes in patients with schizophrenia were found to be significant in a wide area of the PFC relative to healthy controls, and patients with schizophrenia with the COMT Val/Val genotype had significantly more reduced NIRS signal changes than the Met carriers in the frontopolar PFC. These suggest that the COMT Met allele might be one of the influential factors in the evolution of the human hierarchical PFC and one of the protective factors in the development of prefrontal cognitive dysfunction in schizophrenia.

### Effect of COMT genotype on neuroimaging signals

The overactivation response in fMRI BOLD signals and PET cerebral blood flow data in the Val/Val genotypes relative to the Met/Met genotypes (hyperfrontality) have been reported and explained as inefficient prefrontal function in patients with schizophrenia [7], [49], [53], [62]. On the other hand, because the current study as well as previous NIRS studies with the verbal fluency task have found underactivation of NIRS signals (hypofrontality) in schizophrenia patients relative to healthy controls [21], [22], it would be appropriate to interpret the attenuation of NIRS signals during the verbal fluency task as the deterioration of PFC function. Therefore, it is reasonable that the Met carriers with schizophrenia had larger prefrontal activity as measured by NIRS than that in the Val/Val individuals in the current study. Nevertheless, owing to the diminished regulatory effects of dopamine and reduced cortical signal-to-noise ratio [63], it has been speculated that patients with schizophrenia and the Val allele may be predisposed to dysregulation of local gamma-aminobutyric acid (GABA) and glutamate transmission in the PFC [64], which might lead to the suggestion that either ‘hyperfrontality’ or ‘hypofrontality’ may be associated with prefrontal abnormality in patients with schizophrenia [62]. In this study, both healthy controls and schizophrenia patients with Val/Val genotype had wider standard error bars (Figure 2), possibly because of the diminished regulatory effects of dopamine and reduced cortical signal-to-noise ratio [63], [64].

### Potential application to clinical evaluation and translational approach

We have also attempted to examine the clinical usefulness of this noninvasive neuroimaging technique. Multichannel NIRS enables the noninvasive and less-demanding measurement of the spatiotemporal characteristics of brain cortical function, which was able to detect the impact of COMT variation in the PFC in this study. Previous studies [19], [20], [65] showed that the COMT Met genotype is associated with greater improvement of memory performance and negative symptoms in patients with schizophrenia treated with antipsychotic drugs. In the present study, the antipsychotic medication dose of Val/Val individual was not significantly higher than that of Met carriers (p = 0.09) or Val/Met individuals (p = 0.11). However, the present findings leave the possibility that patients with the COMT Met genotype respond more favorably to antipsychotic treatment and that Val /Val individuals require a higher medication dose for being clinically stable. Beltolino et al [19] suggested increasing prefrontal levels of dopamine induced by antipsychotics might have a greater impact in Met/Met individuals, possibly because the effect lasts longer or crosses a threshold of normal range (Figure 4) in dopamine signaling that positively modulates intrinsic prefrontal processing. Taken together, prefrontal hemodynamic response assessed by NIRS could help to predict such effects before starting the treatment and might be useful for the evaluation and prediction of medicinal benefits. Therefore, this study not only replicated the association between COMT genotype and prefrontal function in previous studies, but also showed the usefulness of NIRS in future research using translational approaches.

Imaging genetics studies have shown that brain function by neuroimaging as an intermediate phenotype could be a more sensitive tool to understand how neurobiology bridges the gap from genes to behavior and psychiatric symptoms than the gene itself as shown by association studies [66]. Although the possibility of false positive findings has been a concern when genetic variants (SNPs) of uncertain functional relevance are related to imaging data in imaging genetics study, Meyer-Lindenberg *et al*. [67] provided the empirical data on false positive rates that the type I error was well controlled in imaging genetics and indicated that positive neuroimaging findings in imaging genetics paradigms point toward positive neurofunctional effects that merit further study using translational approaches. Further extensive longitudinal study using translational approaches will be required in the near future to investigate the relationship between the NIRS signals and the improvement of clinical characteristics with antipsychotic medication. Fortunately, NIRS measurements have several beneficial features, being noninvasive, portable, low in running cost, less restraining and less demanding for the participants compared with other imaging methods. These characteristics make NIRS highly desirable for application to repeated clinical evaluations of individual patients.

### Limitations

Some limitations of this study should be pointed out. First, because the 45 participants with schizophrenia were all medicated (Met carriers, only first-generation antipsychotic drugs: 13 patients, only second-generation antipsychotic drugs: 4 patients, both drugs: 8 patients; Val/Val individuals, only first-generation antipsychotic drugs: 8 patients, only second-generation antipsychotic drugs: 5 patients, both drugs: 7 patients; chi-square test, df = 2, p = 0.66, n.p.), we cannot fully exclude the possibility of medication effects, although there was no significant correlation between dose of medication (chlorpromazine equivalent dose, biperiden equivalent dose, and diazepam equivalent dose) and hemodynamic response at any channel. Investigations into drug-naive or drug-free patients are an important next step. Second, the demographic results between normal controls and patients with schizophrenia showed the significant difference in education, socioeconomic status, estimated premorbid IQ, and task performance. As described in Methods section, we gave priority to match these factors for the two genotypes in each diagnostic group and we did not match for these factors between normal controls and patients with schizophrenia, because matching for all the factors lead to the drastic reduction in number of the subjects in each genotype. In fact, these factors were not significantly correlated with NIRS signals at any channel in either group. However, imaging genetic studies in demographically matched samples between normal controls and patients with schizophrenia are also ideal. Third, the results of functional MRI and PET studies during n-back task differed from those of this NIRS study regarding prefrontal regions and signal direction of the impact of the COMT genotype. For an extensive investigation, a replication study using the n-back task with NIRS is required. Fourth, some researchers placed a high value on [deoxy-Hb] decrease due to cognitive activation [25]. However, variances in [deoxy-Hb] were too small to detect the impact of the COMT genotype in patients with schizophrenia in this study. A different prefrontal task with larger effects on [deoxy-Hb] should be employed to further study the effect of the COMT genotype on prefrontal function.

### Conclusions

In conclusion, an implication of the impact of the COMT genotype on prefrontal cortical function in patients with schizophrenia was also identified by noninvasive and less-demanding NIRS measurement. The evidence of association between COMT genotype and increasing risk for developing schizophrenia remains to be elucidated; however, the results of this study suggested that NIRS that is sensitive to COMT genetic variation in patients with schizophrenia might be a noninvasive tool and that prefrontal hemodynamic response may be a useful index for the evaluation and prediction of clinical response such as improvement of prefrontal cognitive dysfunction by atypical antipsychotic medication in clinical settings. This study not only replicated the effect of COMT on prefrontal cognitive function in schizophrenia, but also showed the usefulness of NIRS in future translational research on psychiatric neuroimaging.
